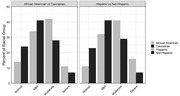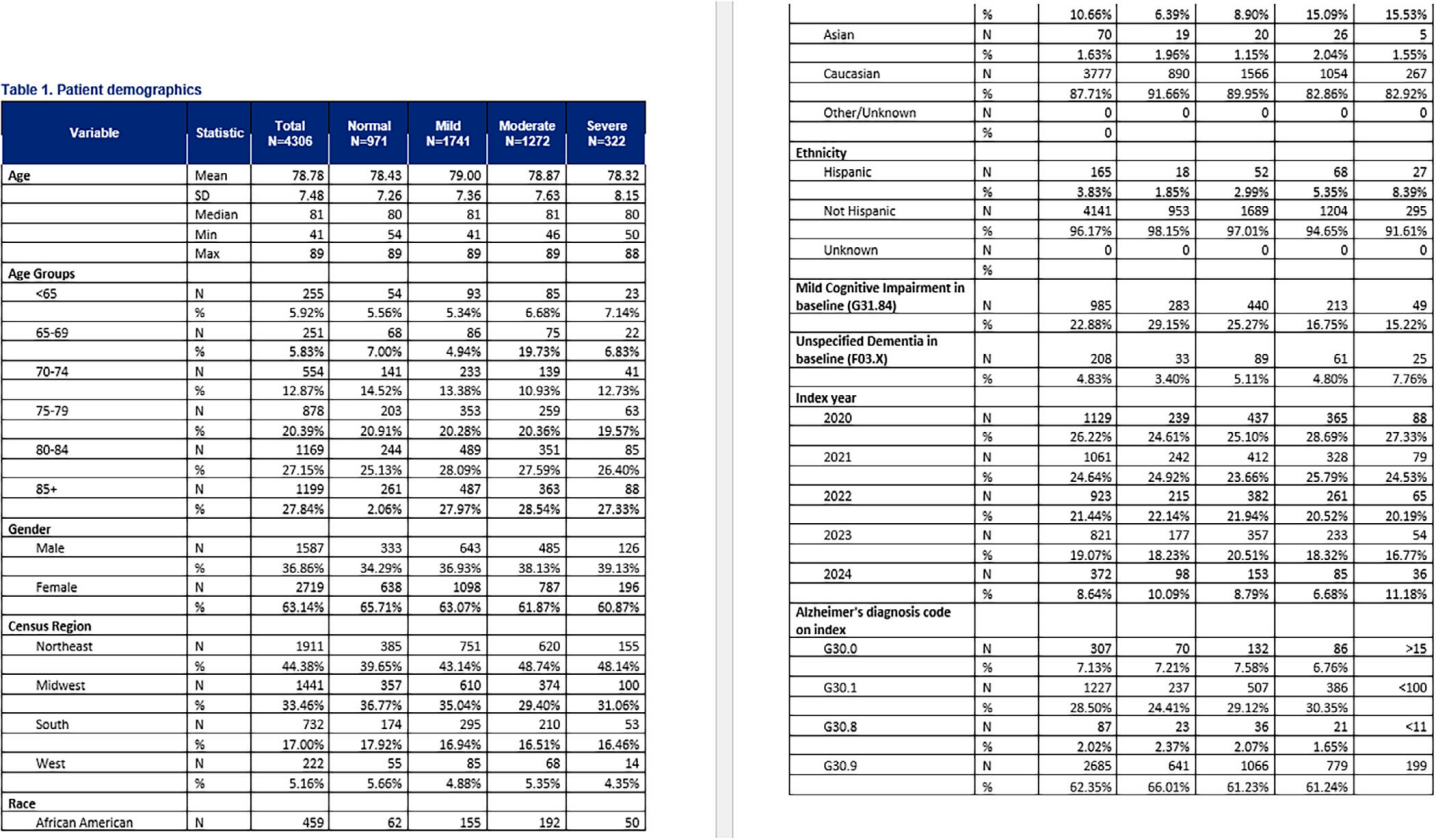# Patient Characteristics, Disease Staging, and Diagnostic Testing Prior Initial Alzheimer’s Disease Diagnosis

**DOI:** 10.1002/alz70861_108435

**Published:** 2025-12-23

**Authors:** William Brady DeHart, Julia M Certa, Jade Pu Zeng

**Affiliations:** ^1^ Optum Life Science, Eden Prairie, MN USA; ^2^ Optum, Eden Prairie, MN USA

## Abstract

**Background:**

The recent developments in Alzheimer’s disease (AD) treatments have elucidated the importance of the timely and accurate diagnosis of AD. As clinical guidelines for the diagnosis of AD are further developed, biomarker testing and neuroimaging will become increasingly important. The purpose of this study was to explore the rates of diagnostic testing in patients newly diagnosed with AD.

**Method:**

This retrospective observational study used de‐identified administrative claims and electronic health records (EHR) from the Optum Market Clarity™ Integrated Clinical + Claims Database to identify US adult commercial and Medicare Advantage enrollees with ≥1 claims for AD (first claim=index date) between 01/01/2020 and 09/30/2024. This dataset was further enhanced by the Optum Alzheimer’s Disease Enriched Clinical Database which leverages validated natural language processing (NLP) methods to extract relevant AD data including cognitive test results (Mini‐Mental State Examination [MMSE] or Montreal Cognitive Assessment [MoCA]). All enrollees had ≥360 days of baseline enrollment. Demographics, AD stage (from cognitive assessments), and biomarker/imaging were captured during the baseline period.

**Result:**

4,306 patients met all inclusion criteria: baseline enrollment, cognitive assessment before AD diagnosis, and no AD diagnosis during baseline. Twenty‐three percent of patients had “normal” cognitive test results, 40% had “mild,” 30% had “moderate,” and 8% had “severe.” African American patients were more likely to be diagnosed in the moderate (41%) or severe (11%) stages compared to Caucasian patients (27% moderate, 7% severe). Additionally, Hispanic patients were more likely to be diagnosed in the moderate (41%) and severe (16%) stages compared to non‐Hispanic patients (29% moderate, 7% severe). Finally, a low number of patients received biomarker testing (1% overall) or neuroimaging (3% overall) prior to their AD diagnosis regardless of AD stage.

**Conclusion:**

Enriching claims data with NLP‐derived clinical values added important depth to this retrospective analysis. By identifying cognitive test results, we found racial and ethnic disparities in AD staging preceding a new AD diagnosis. We also found a suboptimal number of patients that received imaging to confirm their AD diagnosis regardless of stage. Future research is needed to better assess the impacts of suboptimal diagnostic testing.